# Anästhesie bei transoraler roboterassistierter Chirurgie

**DOI:** 10.1007/s00101-025-01566-x

**Published:** 2025-07-21

**Authors:** Marlon Jolissaint, Maximilian Marggraf, Dovile Diktanaite

**Affiliations:** 1 Klinik für Anästhesie, Luzerner Kantonsspital Zentrumsspital, Luzern, Schweiz; 2 University of Luzern, Luzern, Schweiz

**Keywords:** Operationsroboter, Atemwegssicherung, Flusskontrollierte Beatmung, Schwieriger Atemweg, Ultradünner Endotrachealtubus, Surgical robot, Airway management, Flow-controlled ventilation, Difficult airway, Ultra-thin endotracheal tube

## Abstract

Die transorale roboterassistierte Chirurgie (TORS) ermöglicht minimalinvasive Eingriffe im Bereich des Mundes, Gaumens, Rachens, Kehlkopfs und der Tonsillen und bietet zahlreiche Vorteile wie verbesserte Präzision, besseren Zugang und geringeres chirurgisches Trauma im Vergleich zu traditionellen, offenen und somit invasiveren Verfahren. Transorale roboterassistierte Operationen stellen jedoch auch neue anästhesiologische Herausforderungen, insbesondere hinsichtlich der Atemwegssicherung, Narkoseführung und des perioperativen Managements. Diese Aspekte bilden den Fokus dieser Übersichtsarbeit. Ein wichtiger Punkt bei TORS ist die Atemwegssicherung, da einerseits viele Patienten Risikofaktoren für einen schwierigen Atemweg aufweisen und andererseits das Ziel besteht, den chirurgischen Zugang sowie die Übersicht im Operationsfeld so wenig wie möglich einzuschränken. Für die Atemwegssicherung bei TORS stehen mehrere Möglichkeiten zur Verfügung. In diesem Artikel werden die Vor- und Nachteile der verschiedenen Verfahren aufgezeigt und besprochen. Dabei gilt ein besonderes Augenmerk einem neuartigen sehr dünnen Endotrachealtubus namens Tritube© (Ventinova, Eindhoven, Niederlande), welcher dank seiner schlanken Form neue chirurgische und anästhesiologische Möglichkeiten eröffnet. Die damit verbundene flusskontrollierte Beatmung (FCV) wird diskutiert und mit etablierteren Beatmungsformen wie der volumen- und druckkontrollierten Beatmung (VCV/PCV) verglichen. Der vorliegende Artikel soll einen Überblick zur anästhesiologischen Betreuung bei TORS bieten, auf Gefahren aufmerksam machen und Strategien erläutert, um diese zu verringern.

## Abstract (en)

Transoral robot-assisted surgery (TORS) enables minimally invasive procedures in the areas of the mouth, palate, pharynx, larynx and tonsils. It offers numerous advantages such as enhanced precision, improved access and reduced surgical trauma compared to traditional, more invasive techniques; however, TORS also presents new anesthesiological challenges, particularly concerning airway management, anesthesia management and perioperative care. These aspects are the focus of this review. A key issue in TORS is airway management as many patients present with risk factors for a difficult airway, while at the same time it is essential to minimize obstruction of the surgical field and access. Several options are available for securing the airway during TORS. This article outlines and discusses the advantages and disadvantages of the various techniques. Special attention is paid to a novel ultra-thin endotracheal tube known as the Tritube©, which thanks to its slim design opens up new surgical and anesthesiological possibilities. The associated technique of flow-controlled ventilation (FCV) is discussed and compared to more established ventilation methods such as volume-controlled ventilation (VCV) and pressure-controlled ventilation (PCV). This article aims to provide an overview of anesthesia management in TORS, highlight potential risks and outline strategies to mitigate them.

## Hinführung zum Thema

Mittels transoraler roboterassistierter Chirurgie (TORS) konnte man die Notwendigkeit von transfazialen sowie transmandibulären Zugängen reduzieren und die mit diesen invasiveren Zugängen verbundene Morbidität vermindern [1]. Bei Kopf-Hals-Tumoren ermöglicht TORS im Vergleich zur konventionellen offenen Chirurgie bessere funktionelle Ergebnisse, weniger intra- und postoperative Komplikationen sowie kürzere Krankenhausaufenthalte, bei gleichbleibender Überlebensrate [2]. Die Fortschritte in der Chirurgie erfordern eine entsprechende Anpassung des anästhesiologischen Managements bei Patienten mit Kopf-Hals-Tumoren. Mit der zunehmenden Popularität von TORS wird dieses Thema voraussichtlich zu einem wichtigen Aspekt der Anästhesiologie werden.

## Einleitung

Heutzutage gelten minimalinvasive Operationstechniken meist als Standard und werden im Vergleich zu offenen Verfahren in der Regel bevorzugt. Ebenso ist die minimalinvasive roboterassistierte Chirurgie bereits in vielen Gebieten wie der Prostata- und Kolorektalchirurgie gut etabliert und gewinnt in der Kopf- und Halschirurgie an zunehmender Beliebtheit [3].

Vor der Zeit von Operationsrobotern waren minimalinvasive Operationen an Kopf und Hals nur schwer zu bewerkstelligen, da die Operationsgebiete vielerlei Schwierigkeiten aufweisen. Insbesondere der Oropharynx imponiert mit äußerst engen Verhältnissen in einem hochkomplexen Operationsgebiet. Bei einem transoralen Vorgehen sind der Zugang sowie die direkte Sicht zum kaudalen Oropharynx stark eingeschränkt. Ebenfalls ist das Operationsgebiet weit entfernt, sodass mit langen Instrumenten gearbeitet werden muss, was zu einer Reduktion der chirurgischen Präzision führt [4]. Da dies in Kombination mit der schlechten Sicht und den engen Verhältnissen in einem derart komplexen Gebiet keine optimalen Operationsbedingungen schafft, wurde oft ein transfazialer oder transmandibulärer Zugang gewählt [5]. Operationsroboter können hierbei für transoral durchgeführte Eingriffe klare Vorteile bieten. Zum einen kann die Sicht durch eine Kamera im Operationsgebiet verbessert und zum anderen die chirurgische Präzision erhöht werden, indem ein mögliches Zittern durch den Roboter eliminiert wird [6]. Diese Eigenschaft kommt insbesondere bei Operationen mit langen Instrumenten umso stärker zum Tragen. Frühere mehrarmige Operationsroboter konnten jedoch trotz der genannten Vorteile die Schwierigkeiten des transoralen Zugangs nicht optimal meistern, da die Arme nicht flexibel genug waren und sich gegenseitig beeinträchtigten. Neue Operationsroboter wie der Da Vinci Single Port© (Fa. Intuitive, Sunnyvale, CA, Vereinigte Staaten) können nun diese Schwierigkeiten deutlich besser meistern, da sie nur einen Arm benötigen. Der Da Vinci SP© wurde für urologische sowie Kopf- und Halseingriffe entwickelt und vereint in nur einem Arm eine 360°-Kamera sowie die notwendigen Operationsinstrumente. Dies schafft bessere Platzverhältnisse und verhindert, dass mehrere Arme miteinander kollidieren. Erste Daten zum Da Vinci SP© zeigen im Vergleich zu früheren Modellen eine kürzere Operationszeit bei vergleichbarer Sicherheit, dies dank verbesserter Manövrierfähigkeit und besserer Visualisierung [7].

## Präoperative Einschätzung des Atemwegs

Viele Patienten, die eine Anästhesie für eine TORS benötigen, weisen Risikofaktoren für einen schwierigen Atemweg auf. Daher ist eine sorgfältige präoperative Beurteilung des Atemwegs auf der Basis einer umfassenden Anamnese und der Sichtung von Röntgenbildern oder Voruntersuchungen wie Nasen- und Panendoskopie unerlässlich. Insbesondere bei Tumoren im Bereich der Atemwege oder nach chirurgischen Eingriffen und Bestrahlungen kann die veränderte Anatomie eine Herausforderung darstellen.

Neben klinischen Parametern wie Mundöffnung, mandibulärer und zervikaler Beweglichkeit, Mallampati-Score, thyreomentalem und sternalem Abstand oder Halsumfang sollte der Einsatz von Ultraschall zur Beurteilung der Atemwege erwogen werden. Die Untersuchung der Atemwege mittels Ultraschall bietet eine strahlungsfreie, kostengünstige Alternative zu CT und Röntgen, liefert präzise Messwerte und kann zur Identifizierung einer schwierigen Atemwegssicherung hilfreich sein [8]. Parameter wie Haut-Hyoid-Distanz, Zungenvolumen oder Haut-Epiglottis-Distanz dienen dabei als prädiktive Faktoren für eine schwierige Laryngoskopie und Maskenbeatmung [9].

Die klassische transnasale Videoendoskopie (TVE) ist eine Standarddiagnostikmaßnahme bei Patienten mit Verdacht auf ein Larynxkarzinom und ermöglicht die Beurteilung der oberen Atemwege und der Trachea, einschließlich Verengungen, Tumoren oder Ödemen [10]. Da bei vielen Patienten, welche eine TORS erhalten, bereits eine TVE durchgeführt wurde, können bestehende Bilder und Videos zur besseren präoperativen Einschätzung des Atemwegs beigezogen werden. Ein extra dafür entwickelter und validierter TVE-Score stellt in Kombination mit dem Mallampati-Score eine nützliche Ergänzung zur herkömmlichen Risikostratifizierung im Atemwegsmanagement dar [11]. Das evidenzbasierte und ebenfalls validierte Tool „Expect-It“ bezieht Informationen aus TVE, früheren Intubationen sowie relevanten Symptomen mit ein, womit es als Entscheidungshilfe für kameraassistierte und Wachintubationen dienen kann [12].

Die moderne Alternative dazu ist die virtuelle Endoskopie – eine CT-basierte 3D-Simulation der Atemwege vom Oropharynx bis zur Carina. Sie verbessert die Interpretation von 2D-CT-Bildern, erhöht die diagnostische Genauigkeit und unterstützt eine sichere Planung des Atemwegsmanagements, insbesondere bei Kopf-Hals-Erkrankungen [13].

Nach Beurteilung des Atemwegs sollte gemeinsam mit dem Chirurgen ein Plan zur Sicherung des Atemwegs erstellt werden. Hierbei sind sowohl der Plan A als auch Alternativen zu besprechen, der Tubustyp, die Tubuslage sowie der Extubationsplan zu berücksichtigen. Da sich der Atemweg durch die laufende Operation, z. B. im Rahmen von Schwellungen, Blutungen oder Gewebsresektionen, erheblich verändern kann, ist eine kontinuierliche Beurteilung des Atemwegs sowohl intraoperativ als auch postoperativ erforderlich, um den erstellten Atemwegplan ggf. anzupassen. In Tab. 1 sind die wichtigsten Vor- und Nachteile der verschiedenen Atemwegssicherungen zusammengefasst.

**Tab. 1:** Vor- und Nachteilen der verschiedenen Atemwegssicherungen

	Vorteile	Nachteile/Risiken
Nasale Intubation	Gute Platzverhältnisse für einen transoralen Zugang Kleines Risiko einer Tubusdislokation	Eingeschränkte Platzverhältnisse für Eingriffe am Larynx aufgrund der Tubusdicke Blutungen durch Verletzungen der Nasenschleimhaut [14] Sinusitis [16] Druckstellen an Nase und Nasenscheidewand [17]
Orale Intubation	Vertrautes Verfahren mit häufig verwendetem Material	Schlechte Platz- und Sichtverhältnisse für einen transoralen Zugang
Tracheotomie	Optimale Platzverhältnisse für einen transoralen Zugang Freies Operationsfeld Umgehung möglicher Engstellen des oberen Atemwegs bei schwierigen Intubationsverhältnissen Geringes Dislokationsrisiko Einfache Atemwegssicherung bei postoperativen Schwellungen oder Blutungen	Zusätzliches Risiko von Blutungen, Infektionen und Verletzungen von benachbarten Strukturen (z. B. Schilddrüse) Höhere Rezidivrate bei präoperativer Tracheotomie [19]
Nasale Intubation mit Tritube	Sehr gute Platzverhältnisse für einen transoralen Zugang Atemwegsengstellen können dank dünnem Tubus besser überwunden werden Eingriffe am Larynx dank dünnem Tubus besser durchführbar	Schulungen des Personals für den Umgang mit dem Tritube und der Beatmung mit FCV sind erforderlich
Jet-Ventilation	Gute Platz- und Sichtverhältnisse im Operationsfeld Niedriger Atemwegsdruck Minimale Atemwegsbewegungen	Kein Aspirationsschutz Bei längerer Anwendung Anstieg der Beatmungskomplikationen [27] Gefahr von Barotrauma durch glottisnahe Engstellen [29] Mögliche Bewegung der Stimmbänder Risiko der Hyperkapnie mit respiratorischer Acidose [28]
ECMO	Optimale Platzverhältnisse für transoralen Zugang Freies Operationsfeld Oxygenierung und Decarboxylierung über ECMO	Sehr invasiv mit einer Vielzahl möglicher Komplikationen [31] Spezielles Material und spezifisch geschultes Personal erforderlich Hohe Kosten

## Verfahren zur Atemwegssicherung

### Nasotracheale Intubation

Ein häufig angewandtes Atemwegsmanagement bei TORS ist die nasotracheale Intubation. Dabei werden Endotrachealtuben mit einem Innendurchmesser von 6–6,5 mm verwendet. Die nasale Intubation bietet gegenüber der oralen Intubation einen deutlichen Platzvorteil im Mundbereich, was den chirurgischen transoralen Zugang in der Regel einfacher und übersichtlicher gestaltet. Dadurch, dass der Endotrachealtubus durch die Nase und den Nasopharynx eingeführt wird, wird er zusätzlich in seiner Position gehalten, was das Risiko von Tubusbewegungen oder -dislokationen verringert. Trotzdem sollte bei nasaler Intubation der Tubus ausreichend tief platziert werden, da eine TORS oft in einer stark reklinierten Position durchgeführt wird und man beim Operieren auf Stimmbandebene keine Cuff-Schädigung riskieren möchte; was insbesondere bei Operationen am Larynx zu beachten ist. Daher muss bei der Auswahl der verwendeten Endotrachealtuben für die nasale Intubation auf eine ausreichende Länge geachtet werden. Das Vorhandensein von Polypen, einer Nasenseptumdeviation oder Verengungen der Nasengänge kann eine nasale Intubation deutlich erschweren. Da das Risiko einer Blutung durch Verletzungen der Nasenschleimhaut besteht, ist auf eine gute Vorbereitung der Nasenschleimhaut zu achten, um dieses Risiko zu senken [14]. Dabei kommen oft Substanzen wie Xylometazolin, Co-Phenylcain (Lidocain 5 % + Phenylephrin 0,5 %) oder eine Mischung von Lidocain und Kokain zum Einsatz [15]. Weitere Risiken der nasotrachealen Intubation bestehen darin, eine Sinusitis oder Druckstellen an Nase und Nasenscheidewand zu verursachen, weshalb auf eine gute Polsterung des Tubus zu achten ist [16, 17].

### Orale Intubation

Die orale Intubation mit einem herkömmlichen Endotrachealtubus wird für eine TORS selten verwendet, da durch den Tubus ein Großteil des Operationsgebietes verdeckt wird und der chirurgische Zugang somit deutlich eingeschränkt ist. Die Verwendung von dünneren herkömmlichen Endotrachealtuben mit einem Innendurchmesser von 4,5–5,5 mm kann bei herkömmlichen Beatmungsformen zu einer dynamischen Hyperinflation führen, da es zu einem Anstieg des intrinsischen endexspiratorischen Drucks (PEEPi oder autoPEEP) kommen kann, was zu einer Verschlechterung der Beatmung sowie der Hämodynamik des Patienten führen kann [18]. Falls doch eine orale Intubation für eine TORS erfolgt, wird oft ein RAE-Tubus verwendet, da dieser möglichst nah der Zunge aufliegt und direkt nach Austritt aus dem Mund gegen kaudal abbiegt, was eine optimale Ableitung der Beatmungsschläuche gegen kaudal ermöglicht.

### Wachintubation mit flexiblem Intubationsendoskop

Eine Intubation beim wachen oder sedierten Patienten mittels flexiblem Intubationsendoskop soll immer dann angestrebt werden, falls eine schwierige endotracheale Intubation sowie eine schwierige bis unmögliche Maskenbeatmung erwartet werden. Dies kann z. B. der Fall sein bei Patienten mit supraglottischen oder glottischen Tumoren, welche eine teilweise Verlegung der Atemwege bewirken, oder bei Patienten mit relevant verminderter Mundöffnung. Bei der Durchführung ist auf eine gute topische Anästhesie des Atemwegs zu achten und darauf, dass die Spontanatmung bis zur korrekten Platzierung des Endotrachealtubus erhalten bleibt, weshalb eine zu tiefe Sedierung zu vermeiden ist [8].

### Tracheotomie

Die Tracheotomie ist die invasivste Atemwegssicherung, welche selbst einen chirurgischen Eingriff darstellt und mit Risiken wie Blutungen, Infektionen oder Verletzungen der umliegenden Strukturen wie z. B. der Schilddrüse verbunden ist. Die Tracheotomie wird häufig bei Patienten mit Larynxkarzinom im Rahmen einer partiellen oder totalen Laryngektomie durchgeführt. Eine präoperativ durchgeführte Tracheotomie bietet für die TORS optimale Platzverhältnisse, da sich kein Endotrachealtubus im Oropharynx befindet. Jedoch zeigen sich auch negative Aspekte einer präoperativen Tracheotomie, sodass man heutzutage dazu tendiert, die Tracheotomie intraoperativ – nach erfolgter Laryngektomie – durchzuführen. Es konnte gezeigt werden, dass bei den Patienten, die präoperativ tracheotomiert wurden, häufiger Rezidive auftraten, insbesondere wenn die Tracheotomie vor einer initialen Radiochemotherapie durchgeführt wurde [19].

### Intubation mit Tritube© und flusskontrollierte Beatmung

Der Tritube© ist ein extrem dünner Endotrachealtubus mit einem Außendurchmesser von 4,4 mm und einem Innendurchmesser von 2,4 mm [20, 21]. Er verfügt über ein Beatmungslumen und ein Lumen zur intratrachealen Druckmessung im distalen Bereich des Tubus. Aufgrund seines schmalen Designs erfordert der Tritube© eine aktive Absaugung, um Air Trapping und Auto-PEEP zu vermeiden. Er darf daher nur mit dem speziellen Evone-Gerät (Ventinova, Eindhoven, Niederlande) oder dem manuell betriebenen Ventrain-Spirometer (Ventinova, Eindhoven, Niederlande) verwendet werden, und es bedarf einer total intravenösen Anästhesie, da eine Inhalationsnarkose mit Evone bauartbedingt nicht möglich ist [21]. Ein herkömmliches Beatmungsgerät muss weiterhin für die Ein- und Ausleitung der Narkose zur Verfügung stehen, da mittels Evone ausschließlich eine kontrollierte Beatmung möglich ist.

Die zugelassene Beatmungsform für den Tritube© ist die Flow-Controlled Ventilation (FCV). Diese erzeugt einen stabilen Gasfluss in und aus der Lunge des Patienten mit einem konstanten Inspirations- und Exspirationsverhältnis von 1:1 [21]. Im Gegensatz zu den meisten anderen Beatmungsformen erfolgt die Exspiration kontrolliert und linear, wodurch abrupte intrathorakale Druckabfälle vermieden und die Bildung von Atelektasen vermindert werden können [21, 22]. Studien an gesunden Probanden haben gezeigt, dass die FCV im Vergleich zur volumenkontrollierten Beatmung (VCV) eine verbesserte Lungenbelüftung, eine verminderte Atelektasenbildung und eine höhere arterielle Oxygenierung bei gleichbleibendem PEEP und PIP sowie reduziertem Atemminutenvolumen ermöglicht [22].

Durch die kontrollierte Exspiration und intratracheale Druckmessung sowie die daraus resultierenden Vorteile bietet FCV mit Tritube aktuell das sicherste Verfahren für die kontrollierte Beatmung durch kleine Lumina.

Die Hypothese, dass FCV einen neuen Ansatz für eine lungenschonende Beatmung darstellen könnte, sollte in weiteren Studien überprüft werden [23]. Beim Acute Respiratory Distress Syndrome (ARDS) zeigte sich bei Schweinen nach der Beatmung mit FCV dünnere Alveolarwände, eine geringere Zellinfiltration und eine höhere Konzentration an Surfactant-Protein A im Vergleich zur Beatmung mit VCV, was auf eine abgeschwächte Lungenschädigung hindeutet [24]. Bei beatmeten Patienten nach Herz-Thorax-Operationen führte FCV auf der Intensivstation zu einer Reduktion der mechanischen Energie und zu einer verbesserten Ventilation der abhängigen Lungenregionen im Vergleich zur druckkontrollierten Beatmung (PCV), wodurch ein stabiler Gasaustausch mit kleinerem Atemminutenvolumen erreicht werden konnte [25].

**Abb. 1:**
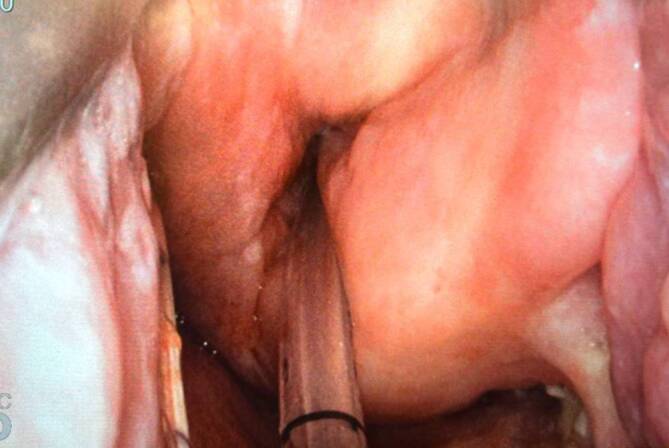
Intubation mit Tritube©

Im Rahmen der TORS-Chirurgie, insbesondere bei Single-Port-HNO-Operationen mit rein transoralem Zugang, bietet der Tritube entscheidende Vorteile. Sein extrem kleiner Außendurchmesser ermöglicht ein besseres Platzangebot und eine freie Sicht auf die Glottis, was in Abb. 1 und 2 zu erkennen ist. Dies kommt insbesondere bei Eingriffen am Larynx und an der Trachea zum Tragen. Die flussgesteuerte Beatmung sorgt zudem für niedrige Flussraten und ein ruhiges Operationsfeld, was die Bedingungen für den Chirurgen optimiert. Der Tritube© ist derzeit der dünnste zugelassene Beatmungstubus für Erwachsene und kann ab einem Körpergewicht von 40 kg eingesetzt werden. Mit einer Länge von ca. 45 cm ist sowohl eine orale als auch eine nasale Intubation problemlos möglich.

**Abb. 2:**
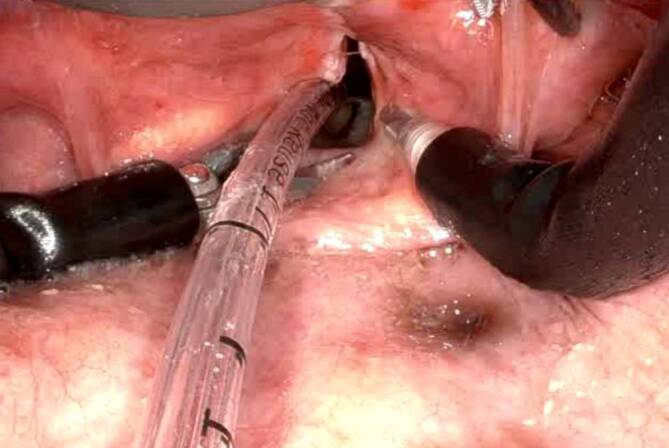
Operation mit Da Vinci Single Port© und Intubation mit Tritube©

Die Intubation wird wie üblich am schlafenden Patienten durchgeführt. Bei Bedarf kann eine Magill-Zange verwendet werden, um den Tubus korrekt im Pharynx zu positionieren. Für die fiberoptische Intubation ist ein alternatives Vorgehen erforderlich, da selbst das kleinste pädiatrische Bronchoskop für den Tritube© zu groß ist. Die Autoren empfehlen hier die Anwendung der Seldinger-Technik: Nach Einführen des Bronchoskops in die Trachea wird ein weicher Führungsdraht über den Arbeitskanal eingeführt, auf den dann der Tritube in Seldinger-Technik aufgesetzt wird. Die korrekte Lage sollte mit einem parallel wieder eingeführten Bronchoskop überprüft werden. Als alternative Vorgehensweise kann eine nasale Intubation unter Spontanatmung mithilfe eines Videolaryngoskops (idealerweise mit hyperanguliertem Spatel) und einer Magill-Zange in Erwägung gezogen werden. Beide Optionen – sowohl dieses Vorgehen als auch die Intubation mittels Seldinger-Technik – erfordern eine entsprechende Personalschulung und ein erfahrenes Team, sind jedoch technisch nicht besonders anspruchsvoll und für die Patientinnen und Patienten in der Regel gut tolerierbar. Perspektivisch könnte die Entwicklung ultradünner Bronchoskope auch eine konventionelle fiberoptische Intubation mit dem Tritube ermöglichen.

Ein Vorteil des Tritube ist die Möglichkeit der Narkoseausleitung bei intubierten Patienten. Der Patient kann aus der Narkose erwachen, während der Tubus in situ verbleibt. Sobald die Spontanatmung einsetzt, kann der Cuff entblockt werden, sodass der Patient am endotracheal platzierten Tritube vorbei spontan atmen kann. Dies bietet zusätzliche postoperative Sicherheit, da der Patient im Falle einer Verlegung der Atemwege durch Schwellung oder Blutung bereits intubiert ist. Eine Spontanatmung durch den Tritube© bei geblocktem Cuff ist hingegen aufgrund des dünnen Innendurchmessers nicht möglich.

### Jet-Ventilation

Eine Jet-Ventilation ermöglicht die Beatmung eines Patienten ohne den Gebrauch eines Endotrachealtubus. Dies kann den Zugang zum Operationsfeld vereinfachen und dem Chirurgen eine bessere Übersicht bieten. Bei der Jet-Ventilation wird ein Katheter oder eine Kanüle vor den Stimmbändern (supraglottisch), zwischen den Stimmbändern (transglottisch), hinter den Stimmbändern (subglottisch) oder durch das Lig. cricothyroideum in der Trachea (transtracheal) platziert. Die Beatmung erfolgt bei der hochfrequenten Jet-Ventilation (HFJV) über kleinvolumige hochfrequente Luftstöße, welche unter hohem Druck durch den platzierten Katheter abgegeben werden. Dabei können Frequenzen bis zu 600 Luftstöße/min verwendet werden [26]. Die Exspiration erfolgt passiv über den offenen Atemweg, da die Trachea nicht mittels Cuff abgedichtet wird. Die Jet-Ventilation ist ein Verfahren für kurze HNO-Eingriffe, welches u. a. im Rahmen der Mikrolaryngoskopie verwendet wird. Für längere Operationen ist die Jet-Ventilation eher ungeeignet, da das Risiko für Beatmungskomplikationen steigt [27]. Insbesondere kann es aufgrund einer inadäquaten Ventilation zu einer Hyperkapnie und konsekutiv respiratorischer Acidose kommen [28]. Im Weiteren kann es bei einer Engstelle im Glottisbereich zu einem erhöhten intrathorakalen Druck kommen, da die insufflierte Luft nicht mehr ausreichend entweichen kann. Mit zunehmendem Air Trapping steigt das Risiko eines Barotraumas, was zu einem Pneumothorax sowie kardiopulmonaler Instabilität führen kann [29]. Ein weiterer Nachteil der Jet-Ventilation besteht darin, dass durch die Beatmung signifikante Bewegungen der Stimmbänder verursacht werden können, was keine optimalen Operationsbedingungen in diesem Bereich zulässt [20].

### „Extracorporeal membrane oxygenation“

Bei Operationen, bei welchen die Beatmung der Lunge und somit die Oxygenierung oder die CO_2_-Elimination vorübergehend nicht in ausreichendem Maße möglich ist, gibt es die Möglichkeit, eine extrakorporale Membranoxygenierung (ECMO) durchzuführen. Dies kann insbesondere bei Patienten mit intrathorakalen Atemwegsstenosen sowie bei komplexen unteren Tracheal- oder Carina-Rekonstruktionen nötig werden, oder falls eine vorübergehende Atemwegsokklusion anderweitig zu erwarten ist [30]. Da die vollständige Oxygenierung und Decarboxylierung außerhalb des Körpers stattfinden können, ist eine Beatmung der Lungen nicht mehr notwendig. Dies ermöglicht ein uneingeschränktes Operationsgebiet mit bestmöglichen Platzverhältnissen und verhindert ein allfälliges Kollidieren von Endotrachealtubus oder Beatmungsschläuchen mit dem Operationsroboter. Der Einsatz von ECMO ist sehr invasiv und mit erheblichen Risiken und Komplikationen (Blutungen, Hämolyse, Nierenschäden, SIRS, Thrombosen, Infektionen, neurologische Schäden) verbunden [31]. Der Einsatz erfordert erhebliche Investitionen in Material, Personal, Expertise und eine ausreichende Anzahl an Fällen pro Jahr und geht mit deutlichen Mehrkosten einher. Aufgrund dieser zusätzlichen Risiken und der genannten Nachteile kommt eine ECMO im Bereich der HNO-Chirurgie nur in wenigen äußerst komplexen Operationen (u. a Carina-Resektionen) zum Einsatz.

## Narkoseführung

### Monitoring

Die TORS wird häufig bei älteren Patienten mit Kopf-Hals-Tumoren und Komorbiditäten eingesetzt. Die komplexen und langwierigen Verfahren können zu erheblichem Blutverlust, Elektrolytverschiebungen und hämodynamischer Instabilität führen. Eine umfassende Überwachung, einschließlich Pulsoxymetrie, EKG, Blasenkatheter, Kerntemperaturmessung und arteriellem Katheter zur Blutdruckmessung sowie Abnahme arterieller Blutgasanalysen, ist für eine optimale Patientenüberwachung unerlässlich [32].

### Aufrechterhaltung der Narkose

Für die Aufrechterhaltung der Anästhesie bei TORS bietet die TIVA gegenüber der balancierten Anästhesie wesentliche Vorteile und wird daher bevorzugt eingesetzt. Die Kombination von Propofol und Remifentanil ermöglicht eine präzise Steuerung der Narkose und eine schnelle Reaktion auf akute Schmerzreize. Bei älteren Patienten ist nach TIVA mit Propofol im Vergleich zu volatilen Anästhetika das Risiko einer postoperativen kognitiven Dysfunktion und eines Delirs reduziert [33, 34]. Außerdem führt Propofol postoperativ zu weniger Übelkeit und Erbrechen, was insbesondere nach einer TORS von Vorteil ist [33]. Während der Operation sollte eine vollständige neuromuskuläre Relaxation sichergestellt und mit einem peripheren Nervenstimulator überwacht werden, um unerwünschte Bewegungen des Patienten, die beim Einsatz von Operationsrobotern schwerwiegende Folgen haben können, zu vermeiden.

### Analgesie

Eine TORS ist dank reduziertem chirurgischem Trauma meist weniger schmerzhaft als die konventionelle offene Operation. Trotzdem kann es postoperativ zu erheblichen Schmerzen, Dysphagie und einem hohen Analgetikabedarf kommen. Da die perioperative Gabe von Opioiden insbesondere bei Patienten mit Nikotin- oder Alkoholkonsum zu einer langfristigen Abhängigkeit führen kann [35], gilt es, Opioide mit Bedacht einzusetzen. Bei opioidnaiven Patienten mit Kopf-Hals-Tumoren liegt die Prävalenz des postoperativ anhaltenden Opioidkonsums bei 18 % [36]. Ebenfalls sollte postoperativ auf eine zu starke Sedierung verzichtet werden, um das ohnehin bereits erhöhte Risiko einer Verlegung der Atemwege nicht weiter zu erhöhen. Um die bestmögliche Analgesie zu erzielen, sollte deshalb eine multimodale Schmerztherapie, mit welcher der postoperative Opioidbedarf gesenkt und die Analgesie verbessert werden können, angewendet werden [37–39]. Mit beispielsweise der zusätzlichen Gabe von Ketamin und Gabapentin konnte im Rahmen einer multimodalen Schmerztherapie der postoperative Opioidbedarf gesenkt werden, bei gleichbleibendem oder verbessertem Schmerzempfinden der Patienten [37]. Es gibt Daten, die zeigen, dass Dexmedetomidin zu einer Verbesserung der postoperativen Schmerzen, Verminderung von postoperativer Agitation sowie einer Verbesserung der perioperativen hämodynamischen Stabilität beitragen kann [40]. Durch die Implementierung von Enhanced Recovery After Surgery (ERAS) konnten bei Kopf- und Halsoperationen postoperativ ein reduzierter Opioidbedarf, eine verbesserte Analgesie sowie ein Verkürzung des Spitalaufenthalts erzielt werden [38, 41]. Durch die intra- und postoperative Verabreichung von Kortikosteroiden bei TORS konnte eine Verbesserung der postoperativen Dysphagie, eine Verkürzung des Krankenhausaufenthalts und eine diskrete Verbesserung der postoperativen Schmerzen erreicht werden [42].

### Flüssigkeitsmanagement

Bei Patienten, welche aufgrund eines Plattenepithelkarzinoms der Mundhöhle eine freie Lappenrekonstruktionen erhalten, ist eine restriktive Flüssigkeitsgabe zu empfehlen. Es konnte gezeigt werden, dass bei diesen Patienten eine erhöhte perioperative Flüssigkeitsgabe mit mehr Lappenkomplikationen, mehr chirurgischen Komplikationen und einem längeren Krankenhausaufenthalt vergesellschaftet ist. Dies traf jedoch nicht auf die intraoperative Verabreichung von Vasopressoren zu. Bei perioperativ restriktiver Flüssigkeitsgabe zeigten sich insgesamt weniger Komplikationen sowie ein kürzerer Krankenhausaufenthalt [43].

## Fazit für die Praxis

- Eine sorgfältige präoperative Beurteilung des Atemwegs ist für eine TORS unerlässlich. Zusammen mit dem Chirurgen sollte ein primärer Plan zur Sicherung des Atemwegs erstellt und mögliche Alternativen besprochen werden.
- Ein häufig angewandtes Atemwegsmanagement bei TORS ist die nasotracheale Intubation. Die nasale Intubation bietet für eine TORS gute Platzverhältnisse im Mundbereich, bringt jedoch auch einige Risiken wie mögliche Blutungen, Infektionen oder Druckstellen mit sich.
- Bei Patienten mit einem Larynxkarzinom sollte eine Tracheotomie erst nach erfolgter Laryngektomie erfolgen, da sie, präoperativ durchgeführt, zu mehr Rezidiven führt.
- Der Tritube© bietet dank seines dünnen Außendurchmessers von 4,4 mm bei TORS eine freie Sicht auf die Glottis, was insbesondere bei Eingriffen am Larynx und an der Trachea von Vorteil ist. Mit einer Länge von ca. 45 cm ist sowohl eine orale als auch eine nasale Intubation beim schlafenden und auch beim wachen Patienten problemlos möglich.
- Die mit Tritube© verwendete Beatmungsform FCV führt im Vergleich zu VCV zu einer verbesserten Lungenbelüftung, einer verminderten Atelektasenbildung und einer höheren arteriellen Oxygenierung bei gleichbleibendem PEEP und PIP.
